# Michigan State Board of Health

**Published:** 1879-06

**Authors:** 


### ARTICLE XI.

MICHIGAN STATE BOARD OF HEALTH. The regular meeting of the State Board of Health was held in Lansing, on Tuesday, April 8, 1879. The following members were present: Dr. R. C. Kedzie, Hon. LeRoy Parker, Rev. D. C. Jacobes, Dr. H. O. Hitchcock, and Secretary Henry B. Baker.

President Kedzie stated that on account of ill health and business connected with oil matters, he had been unable to prepare his annual address. By a vote of the board, he was requested, if convenient, to make his annual address on the subject of the history of legislation relative to illuminating oils in the State of Michigan. The motion also included a request to have in condensed form the main facts bearing upon the dangers in the use of kerosene oil.

Dr. Kedzie was unanimously re-elected president of the board for the ensuing two years.

### SLAUGHTER-HOUSES.

Dr. Kedzie presented the subject of slaughter-houses in towns and rural districts, and read a letter from F. Andrews of Dowagiac, relative to slaughter-houses in his vicinity. Dr. Kedzie referred to the rules which he had prepared for the government of slaughter-houses in the township of Lansing, and suggested the desirability of butchers uniting in one house in cities of even the size of Lansing, for greater ease of methods, for cleanliness and sanitary arrangement. Referred to a committee for investigation and report. This committee is also expected to prepare a model set of rules for the regulation of slaughter-houses.

### DEATH OF REV. MR. BRIGHAM.

The secretary announced the death of Rev. C. H. Brigham, a former member of the board, and proper resolutions of respect to his memory were passed.

### NEW LEGISLATION.

The secretary also announced the passage by the present legislature of three new laws, which will tend to promote the public health in this State and increase the efficiency of local boards of health. Of these laws, one provides that the council of each city and village shall be a board of health, unless there is other provision by special law; so that hereafter there is to be a local board of health in every township, village and city in the State; one law makes it the duty of health officers of cities and villages to notify the prosecuting attorney of any neglect by householders or physicians to report cases of disease dangerous to the public health ; and one authorizes boards of health in cities, villages and townships to furnish free vaccination to the inhabitants thereof. The secretary is to prepare a circular to health officers in cities, townships and villages, calling attention to some of their duties under the new laws, and otherwise setting forth their duties as health officers.

The board adopted a resolution tendering a hearty vote of thanks to Congressman McGowan for his labors in procuring the passage of the bill which has recently become a law, establishing a national board of health.

COMMUNICATIONS WERE RECEIVED

from Hon. C. D. Randall, of Coldwater, suggesting the propriety of the selection of healthy locations and the determining of plans and specifications for all new State institutions, by the board of health, and that all systems of drainage, sewerage, etc., in public buildings hereafter to be erected, should be approved by the State Board of Health previous to their adoption.

Also, from J. A. Russell, M.D., of Edinburgh, Scotland, setting forth a plan for a mutual sanitary protective association for cities, accompanied with a leaflet, giving the plan adopted in that city.

Also, from the national educational bureau at Washington, inclosing a communication from Wm. M. Evarts, Secretary of State, giving notice of a prize of £100 offered by the Royal College of Physicians of London for the best essay on hydrophobia, its nature, prevention and treatment.

Also, a communication from B. B. Ross, of East Saginaw, suggesting that health officers ought to visit all cases reported as dangerous to the public health and verify the diagnosis of the attending physician. He thought this would improve the accuracy of the weekly reports of diseases by health officers. The communication was referred to the committee on legislation, as was also a suggestion by Dr. Baker that the health officer should be authorized to act promptly for the restriction of such diseases, if found to be correctiy reported. Another communication, on the same subject, received from E. S. Richardson, M.D., was referred to the same committee.

At the afternoon session Dr. Lyster was present, and presented an article, portions of which he read, relative to the reclamation of

### OVERFLOWED OR SATURATED LANDS.

Reference was made to large tracts of land on the Crapo farm, the Chandler farm, and near Detroit, and descriptions given of the methods adopted and their results. It also included a record of experiments made for the past twenty years in a large tract of country near Bordeaux, France, translated from a French report. This paper showed the great importance of the work, both pecuniarily and as directly related to health. The original French paper was illustrated with a diagram, showing the relations of the birth-rate to the death-rate as connected with this process of reclamation. It showed an increase of the birth-rate over the mortality until the Franco-Prussian war.

In the discussion which followed, Dr. Kedzie referred to a paper by Judge Albert Miller, of Bay City, on the same subject, giving his experience in the Saginaw valley, where he is reclaiming a section of very valuable land by protecting it with dykes, pumping the water out with steam pumps, and then keeping down the leakage by means of wind-mills. He received the thanks of the board for his paper, and was requested to extend his investigations far enough to include the inspection of overflowed lands in Gratiot county and some other portions of the State, and report upon them, together with recommendations as to what ought to be done.

Dr. Lyster also reported having prepared by request of the board the draft of a plan for a circular on the subject of

### HOUSE-DRAINS AND PUBLIC SEWERS.

The circular is to be studied and elaborated by each member of the board before it is printed and sent out.

Dr. Kedzie said he wished to give an additional point to be considered in testing tin utensils for the presence of lead. His method heretofore published, as thus modified, would be to moisten the tin with nitric acid over a space the size of a dime, dry thoroughly, place thereon a drop of water and then a drop of iodide of potassium. If the tin is adulterated with lead, the spot will assume a yellow color.

In reply to a question as to the

DEATH RATE IN THIS STATE, whether increasing or decreasing under the enlarged sanitary work done in the past, Dr. Baker said it was difficult to speak as regards the whole State, but he brought forward a statement received from Dr. W. H. Rouse, a correspondent in Detroit, giving the total interments in that city since 1873, whereby it appears that while the number of inhabitants has been constantly increasing, the number of interments has been constantly decreasing. The figures given were as follows: Interments in 1873, 2,506; in 1874, 2,386; in 1875, 2,321; in 1876, 2,317; in 1877, 2,105; in 1878, 1,909. He gives for the years 1876–7–8 the number of deaths from each cause, whereby it appears that the decrease has been in certain preventable diseases, rather tending to show that the sanitary work at Detroit in perfecting the water supply and otherwise has resulted in lessening the death rate. In one instance it is almost certain that the lessening of deaths has come from vaccination. In 1877 there were 107 deaths from small pox, and vigorous efforts were made for a thorough vaccination throughout the city. In 1878 not a single death was reported from this cause.

NEW LEGISLATION.

Hon. LeRoy Parker, from the committee on legislation, reported having prepared various bills which had been brought before the legislature, three of which had become laws. One relative to an improved system of holding coroners' inquests, and another in which he had acted with the secretary of the board, in reference to an improved method of collecting vital statistics, is now before the legislature. The memorial for

A SANITARY SURVEY

Had been delayed, owing to lack of time and difficulty of getting the matter properly before the legislature. Mr. Parker and Dr. Baker recommended that the details of a plan be worked out in the board before presenting it two years hence for legislative action; and that there be a committee on sanitary survey, charged with the preparation of schedules for such survey, to which committee all papers or suggestions relating to the subject shall be referred. A committee of three was appointed.

This was the meeting for the reorganization of committees; but as there are vacancies in the board the subject was postponed until the July meeting, A large portion of the work of the board is assigned to committees, and as communications come to the secretary they are forwarded to the proper committees for action. During the quarter, communications had been received and referred to committees, as follows: To Homer O. Hitchcock, M.D., 1, a postal from W. W. Switzer, M.D., relative to three cases of typhoid fever caused by use of bad water; 2, a letter from J. P. Gray, relative to sawdust and mill refuse in streams; 3, a letter from J. D. Johnson, sending a petition against allowing sawdust, etc., to flow into the lake; 4, a letter from C. W. Marvin, M.D., being a study of an outbreak of diphtheria in Gratiot county. To Henry F. Lyster, M.D., 1, a letter from Josiah Miller, relative to flooding Chippewa river for driving logs; 2, a letter from Daniel F. Swain, of Hungerford, relative to cutting away a dam; 3, a letter from W. F. Jenison, of Eagle, relative to drainage, etc.; 4, a letter from J. VanZandt, relative to the dam on Lincoln's Lake.

The secretary, having made some experiments and had some correspondence on the subject of illuminating oils, was requested to turn over to Dr. Kedzie, if he desired it, all correspondence in relation to that subject.

### EXAMINATIONS IN SANITARY SCIENCE.

Dr. Lyster made a report on the proposition, originally made by him, that the board shall offer to examine candidates in sanitary science and its different branches, recommending that the board make preparations for examinations by its different committees on subjects assigned to them, and that certificates be given to those who ask for and sustain examinations. It was thought that the publication of the examination papers would tend to increase the interest and knowledge concerning the subject among the people generally; and that the examinations would tend to secure throughout the State a class of physicians especially intelligent on the subject of sanitary science, and the public could have proof of their qualifications by means of these certificates. If the people see fit to select such persons for health officers, it would react well on the interest of public health, which it is the duty of the board to promote.

Dr. Baker favored it, and suggested that schedules of questions in each of the several branches of sanitary science be prepared for this purpose. The secretary was directed to procure copies of the examination papers in sanitary science from different colleges in foreign countries.

### SANITARY CONVENTIONS.

A communication was received from Dr. Peters, of Tecumseh, inviting the board to hold a convention at that place. The board voted to hold two public sanitary conventions next winter, and each member pledged himself to make them a success. It is desired to procure at these meetings the greatest collection of sanitary utensils which can be obtained, from a common pie-dish to the most elaborate apparatus for heating and ventilation. The time and place for holding such conventions will be announced as soon as determined; and it is hoped that dealers in sanitary appliances will exhibit their wares, and describe their uses and advantages.

Drs. Hitchcock, Lyster and Baker were appointed a committee to prepare for the details of these sanitary conventions.

The secretary presented a report of work done in the office during the quarter. It included the distribution of over 1,000 copies of the Sixth Annual Report, the printing, addressing and mailing of about 2,500 blanks for return of annual reports of health officers and clerks of local boards of health, a large number of which had been received, examined and filed. Circular 29, relative to diseases in Michigan in 1878, had been sent to each correspondent, and replies from 26 persons had been received, examined and filed. Meteorological observations had been taken at the office during the quarter. Meteorological registers and reports of diseases had been received from observers, to whom also the regular distribution of blanks had been made. Work had been done in compiling the weekly reports of diseases, and the meteorological data, for 1878. The correspondence and routine work of the office had been fully up to the average.

The subject of

ANNUAL REPORTS OF HEALTH OFFICERS

and clerks of local boards of health was discussed. It is found difficult to compile these reports in a satisfactory manner by counties, because in many cases one or more townships are not represented by reports. Some officers fail to report, because no reports are made to them; though one effort of the State board is to learn just such facts as the probable extent of delinquencies in the reports of physicians and householders.

The next regular meeting of the board will be held July 8.

---

NERVE STRETCHING.—(*Journn. de Méd.*, Jan., 1879, p. 41.)—Blum practiced this operation in a case of paralysis of the median and radial. A man who a month before had received a cut on the fore-arm, entered the hospital with weakness of arm and pain. Blum exposed the median and radial nerves, elevated them slightly. The next day the muscles began to contract, and the derangements of sensibility had partly disappeared; soon after the patient was completely cured. Duplay reported another case where the troubles of motility and sensibility were due to a tumor situated along the course of the cubital nerve. The diagnosis was neuroma of this nerve, but when operated upon, the tumor was found to be on the tendon of the anterior cubital muscle, and only acted on the nerve by the irritation of its vicinity. The nerve was stretched slightly. The movements returned very rapidly.

THERE will be a meeting of the Illinois State Board of Health, for the transaction of public business, and examination of candidates under the Medical Practice Act, at the Grand Pacific Hotel, Chicago, Thursday, June 12th, 1879, beginning at 8 p.m.

A. L. CLARK, Secretary.

